# The Interactional–Institutional Construction of Teachers’ Emotions in Hong Kong: The Inhabited Institutionalism Perspective

**DOI:** 10.3389/fpsyg.2019.02619

**Published:** 2019-11-26

**Authors:** Kwok Kuen Tsang

**Affiliations:** College of Education Administration, Faculty of Education, Beijing Normal University, Beijing, China

**Keywords:** teachers’ emotions, inhabited institutionalism, institutionalism, interactionism, institutional logics, education reform

## Abstract

This article illustrates the social construction of teachers’ emotions by drawing on the emergent sociological perspective of inhabited institutionalism to report on a qualitative research project on teachers’ emotions in Hong Kong. A thematic analysis was performed on the transcripts of interviews conducted in 2012 with 21 teachers at Hong Kong secondary schools and on the policy documents and newspaper articles from the education reform era of 1980 to 2011. Three major themes emerged from the data: (1) the institutional logic of whole-person education, (2) the institutional logic of accountability, and (3) an asymmetry between these institutional logics, which is causing a displacement of the meaning of education and thus has emotional consequences for teachers. Taken together, these themes show that managerialist education reforms bring the institutional logic of accountability into the institutional environment of education, which results in the recoupling of school administration and teachers’ work. This recoupling leads to the decline of teachers’ work autonomy. The institutional logic of accountability tends to inhibit the institutional logic of whole-person education and to replace the instructional meaning of education with managerial meanings. In the institutional context, teachers are forced to do a lot of work that they interpret as meaningless, but they find that they are powerless to change the situation. They may therefore choose to inhabit the institutional logic of accountability and the tightly coupled institutional context of school organizations. Consequently, teachers may become unhappy at work during and after managerialist education reforms. According to these findings, teachers’ emotions can be regarded as an interactional–institutional construction. That is, teachers’ emotions may be socially constructed through the negotiation of meaning under the institutional logics that guide their actions and the interactions that uphold the institutional context of the school organizations that they inhabit.

## Introduction

The emotions experienced by teachers, such as dissatisfaction, stress, depression, and anxiety, can influence both their effectiveness and well-being in the context of education reforms (Day and Qing, 2009). Education researchers have thus investigated how teachers’ emotions are socially constructed in the context of education reforms to form a framework of recommendations on how to improve their emotions and in turn their effectiveness and well-being (Kelchtermans, 2005; Isenbarger and Zembylas, 2006; Santoro, 2011; Tsang, 2019). According to Tsang and Jiang (2018), institutionalism and interactionism are two sociological perspectives commonly applied to investigate the social construction of teachers’ emotions in the literature. Institutionalism suggests that human emotions are structurally constructed by institutions that regulate the entire social lives of social actors, whereas interactionism argues that emotions are the outcomes of the meaning-making of social actors with limited institutional influence (Turner and Stets, 2005). This article argues that neither of these explanations fully captures the dynamic social construction of teachers’ emotions because emotions should be considered as a social phenomenon co-constructed by institutions and the meaning-making of social actors (Turner, 2007). In this article, understanding of the dynamic nature of the social construction of teachers’ emotions in the context of education reforms is advanced by drawing on the emergent sociological perspective of inhabited institutionalism (Hallett and Ventresca, 2006b; Haedicke and Hallett, 2016). Suggesting that teachers’ emotions are an interactional–institutional construction means that emotions are the outcome of a negotiation of meaning between social actors, in this case school administrators and teachers, under institutional logics that regulate their behavior, which in turn upholds the institutional context that they inhabit.

The aim of this article is to illustrate this point by presenting a study of teachers’ emotions in Hong Kong. To help readers outside Hong Kong to understand the background of the study, the article begins with a brief introduction to the social phenomenon of teachers’ emotions in Hong Kong. Nonetheless, it is noted that the Hong Kong situation is similar to that in many western and eastern countries, where there have been similar trends of education reforms, leading to unhappiness among teachers (Hargreaves, 2003; Chen, 2016; Besley, 2019). In this sense, the findings of the study may also help us understand teachers’ emotions in other countries. After introducing the Hong Kong situation, the article will review the institutional and interactional perspectives on teachers’ emotions and introduce inhabited institutionalism as the study framework before presenting the research method, the findings, and a discussion.

## Teachers’ Emotions in Hong Kong

Hong Kong teachers have been reported as experiencing negative emotions relating to their work, including stress, depression, and anxiety. The Hong Kong Federation of Education Workers (2016) reports that over 80% of teachers in Hong Kong feel stressed and exhausted, over 40% are frustrated, and nearly 30% are unhappy. Cheng (2009) estimates that 50% of teachers in Hong Kong feel powerless in their teaching, over 25% are depressed and anxious, and between 37 and 56% have considered resigning from the profession. According to Lee et al. (2007), the percentage of teachers in Hong Kong that suffer from anxiety and depression is two to three times higher than that of the general public.

In Hong Kong, there is a general belief that the negative emotions experienced by teachers relate to the education reforms that have been implemented since the 1980s. Since that time, the government has issued a series of policy documents encouraging schools to promote students’ all-round development alongside their academic training, through guidance work, moral and civic education, and extracurricular activities. As in the United States, the United Kingdom, and Australia, the government began to reform the education system based on market logics and a managerial approach (Mok and Welch, 2002).

In the early 1990s, the colonial government implemented a pilot project called the school management initiative (SMI) to reform the school governance system based on an educational accountability framework. Since 2000, the government of the Hong Kong Special Administrative Region (HKSAR) has required all schools to implement school-based management (SBM) to promote educational accountability, effectiveness, efficiency, and economy. To enable students to develop adaptability, creativity, independent and critical thinking, and lifelong learning capabilities, the HKSAR government initiated the Learning to Learn curriculum reforms in 2001 and the New Senior Secondary curriculum reform in 2009. These reforms changed the academic structure of secondary schools, promoted formative and school-based assessment, reduced the two high-stakes public examinations to one, and introduced new subjects to the curriculum, including Liberal Studies and Other Learning Experiences.

Researchers observe that these reforms tend to reduce Hong Kong teachers’ autonomy and freedom while intensifying their workload, resulting in negative emotions among teachers (Lee et al., 2007; Chan, 2011). To explain these negative emotions, many scholars analyze the phenomenon from the perspective of institutionalism.

## Institutionalism

Institutionalism is a sociological perspective that investigates the institutional patterns of organizations through their relationship with the wider institutional environment in which they are rooted (Hoy and Miskel, 2012). In particular, institutionalism seeks to understand why organizations located in different communities and countries have similar institutional structures and how organizations adapt to changing conditions in the institutional environment (Ballantine and Hammack, 2013). Basically, the theory argues that there are institutional logics, which are the shared cultural ideals guiding and defining the operations of organizations, and that changes in these institutional logics drive changes in organizations (Meyer and Rowan, 1977).

Accordingly, when this perspective is applied to research on teachers’ emotions in the context of education reforms, these emotions are regarded as the result of institutional changes in schools generated by the reforms. Meyer and Rowan (1977) claim that the institutional environment of education has an institutional logic of rationality that legitimizes bureaucracy as the most effective and efficient form of school administration, leading schools, like other organizations, to develop a bureaucratic structure of administration. However, the institutional logic of rationality is mythical to some extent because there is much evidence of the ineffectiveness, inefficiency, and even irrationality of bureaucracy (Hoy and Miskel, 2012). According to Meyer and Rowan (1977), to avoid the negative consequences of bureaucracy, schools have a bureaucratic outlook but are less bureaucratically administered in practice. As such, schools may avoid exercising strict administrative control or conducting close inspections of teachers’ work. School administration has thus traditionally been loosely coupled with teachers’ work (Meyer and Rowan, 1977), providing teachers with freedom and autonomy in performing their teaching roles (Ingersoll, 2003).

Managerialist education reforms, however, change the institutional relationship between the school administration and teachers’ work from loosely coupled to tightly coupled (Spillane and Burch, 2006). Based on market logics, such as accountability, these reforms aim to bureaucratize school administrative practices (Oplatka, 2004). For example, Pang (2002) finds that when an SMI/SBM is implemented, the administrative practices of Hong Kong schools emphasize bureaucratic values, such as organizational hierarchy, bureaucratic control, achievement orientation, and rationality. This process of bureaucratization implies a tight coupling between the school administration and teachers’ work through which teachers’ work becomes subject to administrative control and inspection (Ingersoll, 2003). As Wong (1997) shows, power and authority are centralized at the top of the school hierarchy with the implementation of SMI/SBM, resulting in the disempowerment of teachers. As a result, teachers may become powerless to exercise control over the teaching process, and types of work that they do not value are assigned to them by school administrators (Smyth, 1995). Under these conditions, teachers are prone to negative emotions, such as stress and depression, because they are exhausted by their heavy workload of tasks that lack meaning for them (Hargreaves, 2003).

Accordingly, the social construction of teachers’ emotions is related to changes in the institutional context of schools, which disempower teachers from exercising control over their work, induced by education reforms. However, as Tsang (2019) suggests, teachers’ emotions are not only an outcome of institutional changes but also constructed by the human agency, which is the capability to make meaning and take action. Teachers’ emotions are their consciously experienced feelings about objects, including the self, aroused by a psychological process of meaning-making through the interpretation and appraisal of those objects (Schutz et al., 2009). As institutionalism neglects the importance of human agency, it cannot comprehensively explain the construction of teachers’ emotions. To overcome this limitation of institutionalism, scholars also investigate the social construction of teachers’ emotions from the perspective of interactionism (e.g., Hargreaves, 2001).

## Interactionism

Belonging to the theoretical tradition of George Herbert Mead and his followers, such as Herbert Blumer, interactionism recognizes the role of human agency in the social construction of human emotions with an emphasis on the dynamics of the self (Charon, 2010). According to this perspective, the self is conceptualized as peculiarly able to be both object and subject. This notion of self is influenced by Mead (1934), who sees the self as composed of “me” and “I.” According to Mead, “me” is the social dimension of the self and serves to guide behavior, thought, and feelings in line with societal expectations, whereas “I” represents the agentive dimension of the self and provides agency for social actors to give meanings to objects and monitor their behavior toward objects. Accordingly, the self is a social reference for behavior (for “me” as an object) that social actors use to reflexively monitor and evaluate their behavior (by “I” as a subject) during interactions (Charon, 2010).

Based on this notion, researchers suggest that emotions are aroused by the meanings that social actors give to the self or the situation through psychological processes, such as interpretation, appraisal, or attribution (Denzin, 1984). The literature suggests that teachers’ emotions may relate to their self-understanding (Kelchtermans, 2005) as moral agents who are devoted to their students’ well-being and welfare (Yin, 2016). This self-understanding may be a reference point for teachers in reflexively interpreting and monitoring their behaviors (Kelchtermans, 2005). If they find that they can exercise their moral agency in teaching and thus verify their self-understanding, they will experience positive feelings; otherwise, they will experience negative feelings (Tsang and Jiang, 2018). Given that the research suggests that education reforms tend to create barriers that prevent teachers from verifying this self-understanding, an interactionist approach is able to explain why teachers will generally feel negative about teaching in the wake of reforms (Day, 2011).

Education reforms may also create unfavorable teaching conditions from teachers’ points of view. Teachers’ workloads are intensified during education reforms (Penrice, 2011). In addition to administrative work, the intensification also occurs in instructional and pastoral duties. Tsang (2019) shows that although some of the intensified work may be instructional or have an instructional meaning, teachers may not recognize the instructional meanings of the work in the context of education reforms. Therefore, they may feel negative about performing the work because they believe that they are carrying out tasks that are unrelated to education but are powerless to change the situation.

From the perspective of interactionism, the social construction of teachers’ emotions may relate to how teachers create meaning for themselves, their work, and their teaching conditions. Teachers tend to feel negative about teaching in the context of education reforms because they negatively interpret themselves, their work, and/or the teaching conditions. Nevertheless, the interactionist explanation has a key limitation: its neglect of institutional influences on meaning-making. According to Scott (2008), institutions are the cognitive, normative, and regulative forces guiding how people behave, interpret, and feel in a given setting. In other words, teachers’ emotions are influenced by institutions. For instance, Winograd (2003) observes that the institution of education requires teachers to show love and enthusiasm toward their students and work, be passionate about their subject matter, avoid displaying extreme emotions, and have a sense of humor in the classroom. According to institutional norms, teachers must manage their emotions and tone down their emotional expression (Winograd, 2003; Isenbarger and Zembylas, 2006). Accordingly, if institutions are taken into account, the social construction of teachers’ emotions may be more dynamic than a purely interactionist approach suggests.

## Inhabited Institutionalism

To address the gap left by sociological studies that overlook either institutions or human agency, Hallett and his colleagues (e.g., Hallett and Ventresca, 2006b; Hallett, 2010; Haedicke and Hallett, 2016) propose the perspective of inhabited institutionalism. This perspective seeks to account for the persistence of various institutional patterns within organizations by placing emphasis on social actors’ meaning-making and interactions (Everitt, 2013). Inspired by institutionalism and interactionism, the basic premise of inhabited institutionalism is that there are institutional logics guiding and shaping organizations made up of social actors whose interactions construct meanings in ways that produce and reproduce these institutional logics. Inhabited institutionalism thereby posits that social phenomena are co-constructed by institutions and interactions (Fine and Hallett, 2014).

Based on institutionalism, inhabited institutionalism adopts institutional logic as a key concept. It argues that institutional logics are the cultural ideals symbolically guiding organizations and social actors. However, institutional logic is understood somewhat differently in the framework of inhabited institutionalism. Institutionalism regards institutional logics as macro-cultural ideals that regulate social organizations and social interactions. For instance, the logic of rationality in the institutional environment of education requires schools to develop a bureaucratic structure that conditions how teachers go about their work. Inhabited institutionalists suggest that institutional logics can also be meso-cultural ideals, such as organizational culture, that influence the institutional context of social organizations (Fine and Hallett, 2014). School culture has been shown to be a key factor shaping the features of schooling processes (Cheng, 2000). For example, schools with a strong learning culture will have a more supportive social structure to encourage teachers to engage in continuous learning, and teachers will therefore tend to be more willing to learn (Chrispeels, 1992). In other words, both macro- and meso-cultural institutional logics work to provide guidelines for social interactions and constitute the institutional context of social organizations.

Based on interactionism, in addition, inhabited institutionalism adopts a concern with the micro-foundation of organizations. According to Hallett and Ventresca (2006a, p. 921), “institutions are inhabited by people and their doing.” This implies that institutional logics are not only guidelines for social interactions but also the outcomes of meanings constructed and propelled forward by interactions between social actors (Hallett and Ventresca, 2006b). A study conducted by Everitt (2013) shows that teachers do not passively follow the institutional logics of education, such as its emphasis on standardization of the curriculum and student outcomes and inclusion and engagement for all students; rather, they actively interpret the meanings of these logics and work in a manner based on these interpretations. Therefore, institutional logics are enacted by teachers’ interpretations and actions in schools. Accordingly, inhabited institutionalism suggests that institutional logics are not purely cultural ideals because they can be instantiated on-the-ground activity through negotiation of meaning and become the basis for ongoing organizational lives (Leibel et al., 2018).

As inhabited institutionalism takes both institutions and human agency into account, it has the advantage of offering a more comprehensive understanding of social phenomena (Bechky, 2011). Although it is an emergent perspective, inhabited institutionalism has been applied to various educational phenomena, such as educational policy implementation (DeRoche, 2013), organizational changes in schools (Hallett, 2010), teacher development (Everitt, 2013), and educational leadership (Lowenhaupt et al., 2016). Accordingly, inhabited institutionalism should be applicable to the study of teachers’ emotions. From this perspective, the present study investigates what institutional logics exist in the institutional environment of Hong Kong’s education system in the context of education reforms, how these institutional logics guide and are related to the negotiation of meaning between school administrators and teachers that constitutes the institutional context of school organizations, and how teachers’ emotions are constructed by the institutional logics and the negotiation of meaning.

## Materials and Methods

The data for this article were collected from a broader research project into teachers’ emotions in Hong Kong that was undertaken in 2012 (Tsang, 2014). The research investigated the patterns of Hong Kong teachers’ emotions in the context of education reforms. In-depth interviews and document analysis were chosen as the data collection methods.

### In-Depth Interviews

In-depth interviews were used because they allowed the researcher to gather rich narrative accounts of teachers’ thoughts, perspectives, feelings, actions, and social environments (Silverman, 2006). Through analysis of these narrative accounts, the researcher was able to identify how the teachers defined themselves, how they did their work and interacted with others in school, how they interpreted their school and teaching environment, and how they felt and why they had those feelings.

The interviews were semistructured with informants asked to talk about what kinds of duties they were responsible for at school, their feelings about their work situation, the reasons why they had chosen to teach, and their experiences throughout their teaching careers. Probing questions were asked to clarify incomplete responses and to elicit more information on the teachers’ emotional experiences. The interview protocol is shown in Table 1. Each informant was interviewed once or twice depending on his or her availability, with the interview sessions lasting for 1.5 h on average.

**TABLE 1:** Interview protocol.

(1) What kinds of duties are you responsible for in your school?
(2) Would you mind describing your working conditions?
(3) How do you feel about your work and your working conditions?
(4) Would you mind telling me the reasons why you teach?
(5) Would you mind telling me about some of your emotional experiences at work during your teaching career?

Interviews were conducted with 21 teachers from 10 secondary schools in Hong Kong. They had a range of teaching experiences and taught a variety of subjects. The participants were selected using maximum variation sampling and snowball sampling. In the first stage, six secondary teachers with fewer than 6 years of teaching experience were invited to participate in the study *via* the researcher’s social network. After a preliminary analysis, the researcher wondered whether the findings would be applicable to more experienced secondary teachers. Thus, seven teachers with more teaching experience were invited to participate in the study *via* referrals. As most of these teachers taught language and humanities subjects, such as English, Chinese, Chinese History, and Liberal Studies, further interviews were conducted with teachers of biology, chemistry and integrated sciences, mathematics, business, accounting and financial studies, and tourism and hospitality studies. These participants were also found *via* referrals. The participating teachers worked at a broad range of schools, including public schools (financed and managed by the Education Bureau), aided schools (publicly funded schools run by religious bodies, charitable organizations, fraternity associations, or voluntary agencies), and Direct Subsidy Scheme (DSS) schools (subsidized or assisted by the Education Bureau in the form of capital grants and bought places). The schools ranked from Band 1 (the most prestigious) to Band 3 (the least prestigious and lowest performing) based on the annual academic performance of Grade 7 students. The sampling ended when data saturation was achieved. The informants’ profiles are summarized in Table 2.

**TABLE 2:** Informants’ profile.

**Teacher**	**Teaching experience range**	**Age range**	**Managerial role**	**Contract type**	**School type**
A	<1 year	30–34	None	Contract CM	Band 2 government (School A)
B	6–10 years	25–29	None	Contract CM	Band 2 aided school (School C)
C	1–5 years	25–29	None	Contract GM	Band 2 DSS school (School D)
D	1–5 years	25–29	None	Permanent GM	Band 3 aided school (School E)
E	6–10 years	30–34	None	Contract CM	Band 2 government school
					(School A)
F	1–5 years	25–29	None	Permanent CM	Band 1 aided school (School I)
G	1–5 years	30–34	None	Contract CM	Band 3 aided school (School E)
H	1–5 years	25–29	None	Contract CM	Band 3 aided school (School E)
I	<1 year	25–29	None	Contract CM	Band 3 aided school (School H)
J	11–15 years	35–39	None	Contract CM	Band 3 aided school (School B)
K	6–10 years	30–34	Subject panel head	Permanent GM	Band 3 aided school (School E)
L	11–15 years	35–39	Subject panel head	Permanent GM	Band 3 aided school (School F)
M	11–15 years	30–34	Subject panel head	Permanent GM	Band 3 aided school (School G)
N	6–10 years	35–39	Subject panel head	Permanent GM	Band 3 aided school (School G)
O	11–15 years	35–39	None	Permanent GM	Band 3 aided school (School G)
P	16–20 years	40–44	Subject panel head	Permanent GM	Band 1 aided school (School J)
Q	36–40 years	55–59	Committee/team leader	Permanent SGM	Band 3 aided school (School G)
R	26–30 years	40–44	Subject panel head	Permanent GM	Band 3 aided school (School G)
S	26–30 years	50–54	Committee/team leader	Permanent SGM	Band 3 aided school (School G)
T	26–30 years	50–54	Subject panel head	Permanent SGM	Band 3 aided school (School G)
U	21–25 years	45–49	Committee/team leader	Permanent SGM	Band 3 aided school (School F)

*CM, Certificate Master; GM, Graduate Master; SGM, Senior Graduate Master.*

### Document Analysis

Different patterns of teaching conditions that might have influenced teachers’ emotions in and after 1980s were identified from the interview data. For example, Teacher S, who had 30 years of teaching experience, said, “When I first started my teaching career, I felt much more comfortable than now. I could go home right after school and enjoy my long holiday without any worry about work” and “The [school organizational] structure was much simpler than now. Well … [there were] fewer activities, clubs, and interest groups [for students].” Similar sentiments were echoed by many of the informants with over 25 years of teaching experience. When asked about the reasons for the changes in the conditions, the informants cited the implementation of education reforms in the 1990s. Despite being encouraged to expound on the details and impacts of the reforms, the informants seemed to find it difficult to explain what kinds of reforms took place and how they affected teaching conditions at the time. Therefore, the in-depth interviews did not provide sufficient data for a thorough analysis of the social construction of teachers’ emotions in the context of education reforms. To overcome this limitation, document analysis was used to generate richer historical data with which to investigate the association between education reforms and teachers’ emotions (Bowen, 2009). The study collected and analyzed education policy documents and newspapers published between 1980 and 2011, when most of Hong Kong’s far-reaching education reforms were initiated. These documents and newspapers were also used to triangulate the informants’ accounts about the impact that education reforms had on their teaching careers (Bowen, 2009). Table 3 lists the education policy documents that were collected and analyzed.

**TABLE 3:** Hong Kong education policy documents collected for the analysis.

**Code**	**Government agency**	**Year**	**Document title**
ACSM01	Advisory Committee on School-based Management	2000	Transforming schools into dynamic and accountable professional learning communities: School-based management consultation document
CDC01	Curriculum Development Council	2000	Learning to learn: The way forward in curriculum development
EMBED01	Education and Manpower Branch and Education Department	1991	The school management initiative: Setting the framework for quality in Hong Kong schools
EMB01	Education and Manpower Bureau	2003	Teacher performance management
EMB02		2005	The new academic structure for senior secondary education and higher education–Action plan for investing in the future of Hong Kong
EMB03		2006	Action for the future: Career-oriented studies and the new senior secondary academic structure for special schools
EDB01	Education Bureau	2008	Performance indicators for Hong Kong schools 2008: With evidence of performance for secondary, primary, and special schools
EDB02		2008	The school development and accountability framework: The next phase of continuous school improvement
EDB03		2011	Recommendations on career guidance for secondary schools under the new academic structure
EC01	Education Commission	1984	Education Commission report no. 1
EC02		1986	Education Commission report no. 2
EC03		1988	Education Commission report no. 3: The structure of tertiary education and the future of private schools
EC04		1990	Education Commission report no. 4: The curriculum and behavioral problems in schools
EC05		1992	Education Commission report no. 5: The teaching profession
EC06		1992	School education in Hong Kong: A statement of aims
EC07		1996	Education Commission report no. 6: Enhancing language proficiency: A comprehensive strategy
EC08		1997	Education Commission report no. 7: Quality school education
EC09		2000	Learning for life, Learning through life: Reform proposals for the education system in Hong Kong
EC10		2000	Review of education system: Reform proposals: Consultation document
ED01	Education Department	1981	General guidelines on moral education in schools
ED02		1985	Guidelines on civic education in schools
ED03		1986	Guidance work in secondary schools–A suggested guide for principals and teachers
ED04		1986	Guidelines on sex education in secondary schools
ED05		1996	Guidelines on civic education in schools
ED06		1997	Guidelines on extracurricular activities in schools
ED07		1997	Guidelines on sex education in schools
ED08		2001	School administration guide
ED09		2002	Performance indicators for Hong Kong schools
ED10		2002	Performance indicators for Hong Kong schools: Evidence of performance
HKGS01	Hong Kong Government Secretariat	1981	The Hong Kong education system: Overall review of the Hong Kong education system
VP01	Visiting panel	1982	A perspective on education in Hong Kong: Report

Newspaper articles focusing on education in Hong Kong were obtained from two channels. The first was *WiseNews*, a news clipping database containing articles about Greater China, including Hong Kong, published in newspapers, magazines, and journals from across the Greater China region. Limiting the results to Hong Kong-based newspapers, relevant articles were identified by a keyword search for “education reform,” “curricular reform,” “education system,” “education policy,” “education,” “secondary school,” “school education,” “schooling,” “teaching,” “teacher,” and “curriculum” in both Chinese and English. As *WiseNews* only contained clippings from 1998 onward, the Hong Kong Newspaper Clippings Contents database published by the Hong Kong Catholic Social Communications Office was also used. Titles listed in the education category of this database were searched using the same keywords. Articles returned by these searches were then reviewed and saved if they were deemed to be relevant. In total, 832 news clippings were collected.

### Data Analysis

After data collection, all of the interviews were transcribed. The interview and documentary data were then analyzed with thematic approach using open coding and then focus coding to identify themes in the documents and interview transcriptions (Esterberg, 2002). Throughout the analysis, the coding scheme was continually refined to improve the credibility of the data analysis by comparing incidents in the data with other incidents, incidents with themes, and themes with other themes (Glaser and Strauss, 1967). Three major themes emerged: (1) the institutional logic of whole-person education, (2) the institutional logic of accountability, and (3) asymmetry between institutional logics, meaning displacement, and emotional consequences for teachers.

## Findings

### The Institutional Logic of Whole-Person Education

The findings revealed that there was an institutional logic of whole-person education that was the cultural ideal of education as cultivating the all-around development of students in the Hong Kong institutional environment of education. The logic was articulated in several education policy documents. For example, EC06 stated the aim of school education in Hong Kong as follows:

The fundamental aim of the school education service is to develop the potential of every individual child, so that our students become independent-minded and socially aware adults, equipped with the knowledge, skills, and attitudes which will enable them to lead a full life and play a positive role in the social and economic development of the community. (p. 9)

In EC10, the logic was expressed as follows:

To enable every person to attain all-round development in the domains of ethics, intellect, physique, social skills, and esthetics according to his/her own attributes so that he/she is capable of lifelong learning, critical and exploratory thinking, innovating, and adapting to change; filled with self-confidence and a team spirit; willing to put forward continuing effort for the prosperity, progress, freedom, and democracy of their society, and contribute to the future well-being of the nation and the world at large. (p. 5)

According to the findings, the institutional logic emerged from negotiations over the meaning of quality in education that had been ongoing between the public and the government, especially the British colonial government, since the 1980s. The collected news articles revealed that during the 1980s and the early 1990s, the public called on the government to improve the quality of education to solve youth problems, such as delinquency and a lack of moral and civic awareness, by promoting whole-person education. The government published a series of education policy documents, including CDC01, EDB03, ED01, ED02, ED03, ED04, ED05, ED06, and ED07, to guide schools in providing different kinds of whole-person education activities. The following paragraph extracted from ED03 gives an illustration:

Pupils’ developmental, educational, and personal problems become more and more visible … the public at large are concerned with the increase of disruptive behavior in the classroom, the lack of motivation toward school work as well as adjustment problems manifested by many pupils … the need to promote guidance work in school since most of pupils’ problems can be overcome, or even prevented, through prompt assistance and appropriate advice … initial intervention can be provided and pupils helped to maximize their own potential, acquire acceptable social skills, discriminate right from wrong, develop appropriate values … be better equipped for real life. (pp. 1, 2)

Following the recommendations of these policy documents, schools began to be departmentalized into several teams and committees to work on providing whole-person education through extracurricular activities, moral and civic education programs, and sex education programs (Tsang, 2019). Although the provision of the whole-person education became more institutionalized, there was a lack of administrative monitoring and inspection of teachers’ work on whole-person education during the 1980s and early 1990s. The loose coupling between school administration and teachers’ work was evident from the teaching experiences of late-career informants who had taught for 25 years or more. They said that while schools might have had some teams or committees coordinating the provision of whole-person education, they were not pressured to do the work at that time.

Talking about our work in early days, our school was purely focused on teaching. We didn’t have special concerns about students’ development. It’s really true…only the guidance team existed from the beginning. Many other teams like the moral education team and counseling team did not exist in the past… The guidance team meets and punishes students. There are no other special teams … Nothing else. (Teacher U)

Nevertheless, the loose coupling did not mean that teachers did nothing to provide whole-person education. Indeed, they seemed to perceive whole-person education as an important aspect of education and teaching.

On the one hand, teaching is about imparting knowledge to the students, but on the other hand, teaching is also about mentoring the personal growth of the students. Education is not just a guide of academic achievement but also a channel for nurturing personal growth—that’s whole-person development. Both aspects should be considered. (Teacher S)

Most of the informants said that they tried their best to help students explore and develop their interests and potential so as to nurture their all-around development even when there were no administrative requirements to do so. They viewed themselves as moral agents working for the well-being and welfare of their students.

As for the meaning of morality in this industry. A teacher needs to pay attention to the needs of different students all the time, such as matching suitable extracurricular activities to particular students to help their development. We always need to think about how to do better… For the sake of morality, we would prefer to do right by ourselves whenever we knew what we do might be good for the students. (Teacher N)

Accordingly, the policies introduced the institutional logic of whole-person education to the institutional environment of education and in turn induced changes in the structure of school administration to support the provision of whole-person education. However, as Morris and Scott (2005) note, the institutional logic of whole-person education played a symbolic role in the Hong Kong institutional environment of education. Morris and Scott (2005) indicate that the colonial government treated the introduction of whole-person education as a means to demonstrate its willingness to address educational issues but did not push hard for its implementation to avoid any conflicts that might subvert its legitimacy. Therefore, schools could symbolically implement whole-person education just by developing an administrative structure because they were neither encouraged nor forced to enact the policy by the colonial government. In this situation, the school administration was loosely coupled with teachers’ work. This loose coupling provided freedom and autonomy for teachers. Under these conditions, even though the school administration may not have been seriously interested in working to provide whole-person education, teachers would still be able to work toward whole-person education insofar as they interpreted the work as meaningful.

### The Institutional Logic of Accountability

The findings suggested that accountability also became an institutional logic in the Hong Kong institutional environment of education once SMI/SBM was initiated in 1991. The institutional logic of accountability, which is the cultural ideal of managerial approaches being the best means of achieving quality in education, was expressed by the government in policy documents, such as EMBED01 and EC08. For instance:

There are difficulties under the present framework in ensuring quality of output and evaluating results … the government’s efforts in school education are less effective than they might be as a result of inadequate management structure and process; poorly defined roles and responsibilities; the absence or inadequacy of performance measures; an emphasis on detailed controls, rather than frameworks of reasonability and accountability; and an emphasis on cost control at the margins, rather than cost-effectiveness and value for money. (EMBED01, paras. 2.0, 2.1)

The successful building of quality school culture… with increased transparency of school operations, broadened participation from parents and the community in school management, increased accountability of schools to the public, and the sharing of experience among schools with similar background or within the same quality circle, schools will be expected and thus motivated to improve and continue to strive for excellence. (EC08, para. 1.10)

Once the institutional logic of accountability took hold, the government proposed various managerial measures, such as school self-evaluations, school external reviews, and performance indicators, to make schools accountable for measurable outcomes. Measures of this kind appeared in ACSM01, EMBED01, EMB01, EDB01, EDB02, EC08, ED08, and ED09. As a result, school administration and teachers’ work were recoupled. For example, the informants commented that after the implementation of SMI/SBM, school administrators became enthusiastic about monitoring teaching practices by various inspection measures, such as book inspection (checking how well the teachers marked students’ assignments and tests) and lesson observation (evaluating teaching quality in the classroom). In some cases, the school administrators made teachers accountable for students’ academic performance, using an internal league table that showed how well students in each class had performed in examinations.

Teacher P: My school would announce the examination results of each class for each subject in the school newsletters. You compare your performance with others, even though others do not. If your class’s results are poor, what do you feel? No face! So you cannot sleep well on result announcement days.

Interviewer: Could you describe what you felt?

Teacher P: Let us say…this year, the pass rate of the subject I am teaching was not 100%. One student failed the examination. Oh my God! In a staff meeting, the principal and the subject panel head asked who taught the student and required the teacher to explain why the student failed. Imagine you were in the meeting—what would you feel? So stressful! The only thing you can do is to pray that none of your students fail the examination.

In addition, school administrators began to monitor teachers’ work on whole-person education. The informants said that if they organized a whole-person education activity, the school administrators would require them to submit a proposal explaining how they would ensure the activity’s efficiency and effectiveness and then a report on how the activity had been efficiently and effectively implemented.

We need to write reports and plans. Indeed, we spend a lot of time writing these. For instance, an annual report doesn’t just take us a few hours of writing by the end of the school term, but the whole school term. We have to do evaluations after each activity, such as distributing questionnaires and collecting opinions from the students, teachers, and parents. All of the data collected and all of the evaluation materials should be included along with supporting documents in the reports…We can’t just make up a report. It is such time-consuming and tiring work. (Teacher S)

In some cases, the school administrators quantified and monitored teachers’ work on whole-person education.

I don’t want to quantify my work according to a piece rate. Instead, I want to pursue the goal of quality. However, most of the time, the school just looks at the piece rate…For instance, when we organize activities (e.g., moral and civic education programs, sex education programs, and extracurricular activities), the school administrators may say, “Okay, you will manage four of them.” You know, sometimes we can’t just organize activities at once. I have my own responsibilities… I am frustrated that they insist that I need to organize the four activities. The school does not care about my opinion. (Teacher K)

**FIGURE 1:**
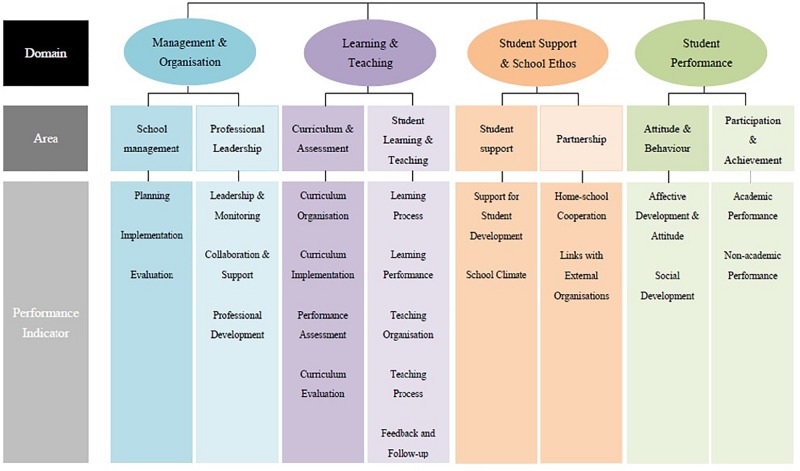
Performance indicators framework. The performance indicators framework is extracted from EDB01, p. 3. It is a three-tier framework, comprising domains, areas, and performance indicators. The government evaluates the effectiveness of schools by focusing on four domains: management and organization, learning and teaching, student support and school ethos, and student performance. Each domain is subdivided into two areas, resulting in eight areas operationalized by 23 performance indicators.

A possible reason for the quantification and monitoring of whole-person education was that this kind of teachers’ work was perceived by administrators as a means to satisfy two domains of performance indicators (Figure 1), “student support and school ethos” and “student performance.” As Teacher L, an informant who worked as a school senior manager, elaborated,

The government has four domains in reviewing a school… the fourth domain is about teachers. The categories of teacher performance, guidance, extracurricular activity, and civil education compose school support and students’ growth (performance). That is, the more activities we organize, the better we can prove that we provide good support for students’ growth (performance). We can make it… because we have a lot of huge goals. Organizing just one or two activities is considered to be insufficient in achieving huge goals. (Teacher S)

This quotation shows how school administrators tended to interpret activities as contributing to the school’s achievement in supporting students’ whole-person development. One possible reason that administrators would make this interpretation is that EDB01 does not provide a concrete articulation of the performance indicators. While EDB01 states that the performance indicator of support for student development is related to several questions, such as “How does the school identify students’ varied needs in the area of support for student development?,” “Is the school’s planning for school-based student support services effective?,” and “Does the school suitably support students with diverse learning needs to help them integrate into campus life and develop their potential?” (p. 22), it does not define clearly how a school would be evaluated as effective or ineffective on the basis of these questions, thus creating a sense of uncertainty among school administrators. Therefore, the school administrators may have preferred to quantify and monitor whole-person education activities to provide objective evidence of their efforts to support students.

Another possible reason for the quantification and monitoring was the quasi-market created by an initiative commonly known as school closure. Under this initiative, schools that could not recruit sufficient students would be forced to close on the basis of being ineffective. To avoid being labeled as ineffective, schools were motivated to compete with one another for student enrollments. Providing more whole-person education activities was regarded as a selling point.

If a school… needs to promote student admission, it usually makes up banners showing how great the academic results and the extracurricular activities are. It’s important to market the clubs at school, emphasizing what awards are attained by certain clubs and sport teams. If a school emphasizes too heavily, it will probably push its students to get more awards. Yet, how do we get more awards? The answer is to push the students to train endlessly and take part in an overwhelming number of activities, to aim high for good results which is helpful in building the school image and promoting student enrollment. (Teacher E)

Accordingly, the institutional logic of accountability tended to recouple school administration and teachers’ work so that teachers became subject to administrative monitoring. To make teachers accountable for measurable outcomes, school administrators monitored teachers’ work through various inspection measures. In this situation, teachers were not able to determine what and how they did their work. The heavy non-instructional workloads placed on them by school administrators resulted in a lack of time and energy for instructional work. Although teachers were dissatisfied with this situation, they were powerless to change it or to reject the assigned work.

All administrative duties are already assigned by the school to me regardless of my willingness. We just can’t say no. We have to get it done. (Teacher A)

This sense of powerlessness might have been stronger among non-tenured teachers.

We’re afraid of expressing opinions because our jobs are contract-based. We’re afraid of being fired. We’re afraid of saying something offensive to others because it might result in the loss of our job. (Teacher B)

Some informants said that they had attempted to negotiate with school administrators and asked them to improve the situation, but the administrators had not acted on their views. The resulting sense of powerlessness made them feel negative about teaching:

I feel quite helpless. I can neither change nor control the reality… I’m not the one who makes the decisions. We are passive. (Teacher M)

### Asymmetry Between Institutional Logics, Meaning Displacement, and Emotional Consequences for Teachers

Since the SMI/SBM reforms, the institutional logic of whole-person education and the institutional logic of accountability have coexisted in the Hong Kong institutional environment in the context of education reforms. However, their relationship has tended to be asymmetric in that the institutional logic of accountability adversely affects the institutional logic of whole-person education. According to the analyzed documents, the institutional logic of accountability has promoted managerial approaches to education as an effective means of improving education quality. The logic of whole-person education seems to have adopted these accountability measures and thus assumed that students’ all-around development could be fostered through measurable activities. However, since the growth of the institutional logic of accountability, the instructional meaning of education quality seems to have become subordinate to the managerial meanings. Ultimately, the instructional meaning of education seems to have been displaced by the managerial meanings in the institutional environment of education. Such a displacement of meaning was observed in the informants’ interpretations of their work.

According to the interview data, the informants often complained that they were overloaded with non-instructional work and thus had insufficient time and energy for instructional work. When asked to give examples of non-instructional work, they frequently cited work related to whole-person education, such as the organization of moral and civic activities, extracurricular activities, and guidance work. Similar observations were found among the collected newspaper articles, which showed that this kind of work was also categorized as non-instructional by the Hong Kong Professional Teachers’ Union, the largest professional teacher association in Hong Kong. Accordingly, even though the work should have instructional value, Hong Kong teachers may perceive it as related to school administration rather than education.

One possible reason for the meaning displacement is that, as implied in the above findings, school administrators treated whole-person education as a managerial means to improve education quality as defined by the performance indicators and/or to promote student admission in the context of the SMI/SBM reforms. Another possible reason is the intensification of administrative duties related to school management attached to whole-person education by the SMI/SBM reforms, which eroded teachers’ time and energy for instructional work. Both situations may have institutionally made it difficult for teachers to find instructional meaning in whole-person education work, associating it instead with managerialism and non-instructional work.

This meaning displacement goes some ways toward explaining the negative emotions of teachers. If teachers do not identify the instructional meanings of the work that is making demand on their time and energy, they may come to dislike the work and be dissatisfied with their work situation because their activities do not match up to, or help them to verify, their self-understanding as moral agents (Kelchtermans, 2005). In this situation, they may question whether they are moral and competent teachers, which could result in a negative self-understanding and thus generate negative emotions.

I feel the current work situation makes me … kind of frustrated. I doubt whether I have done a good job. This is because … now we have to do a lot of work unrelated to teaching. It’s overwhelming… Sometimes I can’t even spare time to prepare my lesson and check students’ assignments …let alone spend time to meet with my students. Sometimes I don’t feel like I am devoting myself to my students … It seems that I’m not a competent teacher. (Teacher D)

Sometimes I feel helpless … The most tragic thing is that I have to make the non-instructional work a top priority. This makes me feel uncomfortable. Like when we organize a big event (e.g., moral education program), I wonder if it is meaningful for the students or just related to the reputation of the school (for promotion). It seems to me that this event, which requires strenuous effort, is not targeting the students. As a teacher … we really want to transfer our academic knowledge or life experience to the students, but does our work link up with our wants? I feel particularly uncomfortable because I have no idea whether the students can learn from the big show on which we have spent tons of effort. What I really want to work on is something that will enable the students to truly learn and grow. (Teacher L)

## Discussion

The literature has suggested that education reforms tend to produce negative emotions among teachers by institutionally recoupling school administration with teachers’ work. This recoupling tends to disempower teachers and subjects them to non-instructional workloads (Smyth, 1995; Penrice, 2011), according to institutionalism, or fails to verify their self-understanding as teachers because it is difficult for them to identify meaningful connections between their work and themselves (Hargreaves, 2001; Kelchtermans, 2005), according to interactionism. The findings of this study, however, imply that these analyses may not fully explain the social construction of teachers’ emotions in the context of education reforms. First, the previous studies were conducted to understand how the negative emotions of teachers are constructed in the context of education reforms. In other words, they may be weak at explaining the social construction of teachers’ positive emotions in the same context (Frenzel, 2014). However, as the findings of this study illustrate, education reforms may not necessarily lead to negative emotions. Hong Kong teachers were prone to negative emotions like stress, frustration, and helplessness in the context of education reforms after the 1980s, while they tended to be less dissatisfied and unhappy in the context of education reforms in the 1980s. In other words, the institutionalist and interactionist analyses may be weak at explaining the patterns of teachers’ emotions in the 1980s in Hong Kong. Second, as Turner (2007) argues, human emotions are co-constructed by institutions and human agency. As the findings indicate, the social construction of teachers’ emotions may involve the interplay between institutional logics, institutional contexts of school organizations, and the negotiation of meanings between social actors. The social construction of teachers’ emotions could thus be more dynamic than institutionalism and interactionism suggest.

To overcome the limitations of existing perspectives, drawing on the perspective of inhabited institutionalism and the research findings, this study suggests that teachers’ emotions be thought of as constructed in a more dynamic interactional–institutional manner. Interactional–institutional construction means that a social phenomenon is socially constructed by the negotiation of meaning among social actors under institutional logics that guide their actions and interactions, which in turn uphold the institutional context that they inhabit (Fine and Hallett, 2014). A change in any of these components may invoke different patterns of teachers’ emotions.

### The Interactional–Institutional Construction of Teachers’ Emotions in the Context of Education Reforms

Teachers’ emotions can be regarded as the function of institutional logics, institutional contexts, and the negotiation of meanings of teachers’ work between social actors. The different patterns of teachers’ emotions identified in the context of education reforms in and after the 1980s help illustrate this observation.

According to Sweeting (2004), there is no significant evidence that negative emotions among teachers were a serious problem during the 1980s. Indeed, the findings of the present study also imply that teachers tended to feel positive, or at least not negative, in teaching during that period of time. According to the findings, the institutional logic of whole-person education tended to dominate the Hong Kong educational system in the 1980s and provided symbolic guidelines for school administrators to institutionalize schools and for teachers to work to improve the quality of education. As the logics tended to match teachers’ self-understanding as moral agents, they were willing to do their best on their own initiative for their students’ well-being and welfare. Meanwhile, the school administrators attempted to institutionalize schools to provide whole-person education according to the guidelines. As the guidelines were symbolic, school administrators were not pressured to manage teachers’ work on the whole-person education (Morris and Scott, 2005), resulting in a loosely coupled institutional context in schools. In such an institutional context, teachers were able to enjoy the autonomy to exercise their moral agency and work in line with their self-understanding, resulting in positive emotions, or at least less negative emotions.

On the other hand, the population of unhappy teachers have increased in Hong Kong since the 1990s (Sweeting, 2004). According to the findings, the institutional logic of accountability arose in the Hong Kong education system, as in other education systems worldwide (Hallett and Meanwell, 2016), with the implementation of SMI/SBM in the 1990s. In contrast with the institutional logic of whole-person education, the institutional logic of accountability is not a mere symbolic guideline but a real pressure exerted on school administrators to manage schools in accordance with managerial approaches (Hallett and Meanwell, 2016). As the findings show, school administrators made teachers accountable for measurable outcomes, resulting in a tightly coupled institutional context. In the institutional context, teachers were subjected to administrative monitoring and inspection and were forced by school administrators to do a great deal of non-instructional work. Teachers felt that this left them with insufficient time and energy to work for their students’ well-being and welfare. Thus, as the literature suggests (e.g., Santoro, 2011), teachers may feel generally dissatisfied with the institutional context. Although some teachers may attempt to negotiate with the school administrators, the findings indicate that they may be powerless to change the institutional context. They may therefore unwillingly and passively choose to submit themselves to the school administration and inhabit the institutional logic of accountability and the tightly coupled institutional context of school organizations. This may explain why the number of unhappy teachers in Hong Kong seems to have increased following the education reforms of the 1990s (Sweeting, 2004).

Based on the inhabited institutional analysis, Table 4 summarizes the differences in institutional logic, institutional context of school organizations, and the negotiation of meanings of teachers’ work in the context of education reforms in and after the 1980s in Hong Kong. Different institutional logics of education reforms have had different influences on the institutional context of school organizations, which are inhabited by school administrators and teachers who negotiate with each other in line with the institutional logics to uphold the institutional context, and in turn construct the social phenomenon of the teachers’ emotions.

**TABLE 4:** Differences in institutional logics, institutional context, and negotiation of meanings between the contexts of education reform in and after the 1980s in Hong Kong.

	**Education reforms in the 1980s**	**Education reforms after the 1980s**
Institutional logics	Logic of whole-person education that values cultivating the all-around development of students and symbolically guides the operation of school organizations and teachers’ work	Logic of accountability that defines managerial approaches as the best means of achieving quality in education and institutionally pressures schools and teachers to do their work with managerial approaches
Institutional context of school organizations	Loosely coupled context in which teachers enjoy freedom and autonomy in doing their work	Tightly coupled context in which teachers’ work is subject to managerial control by school administrators
Negotiation of meanings of teachers’ work	Teachers can exercise their moral agency to define the meanings of their work and do their work in line with their self-understanding	Teachers tend to be powerless to negotiate the meanings and contents of their work with school administrators who define the situation of teaching

### Alienated Institutional Environment of Education

To some extent, the findings imply that education reforms, especially managerialist education reforms, alienate the educational goals from the institutional environment of education but align the managerial goals to it. The alienated institutional environment is reflected by the findings of the asymmetric relationship between the institutional logics of whole-person education and accountability. According to the findings, although the Hong Kong government would like to promote the quality of education by initiating different education reform initiatives, it has preferred to define the quality of education by the logic of accountability rather than the logic of whole-person education since the 1990s. Therefore, accountability becomes the end of education instead of the means to pursue quality of education, whereas whole-person education becomes the means for quality assurance rather than the end of education in the Hong Kong institutional environment (Choi, 2005). In the alienated institutional environment of education, as the findings show, the instructional meanings of teachers’ work may also be displaced by managerial meanings. Thus, it becomes hard for teachers to identify meaningful connections between the work and their moral selves, resulting in a variety of negative emotions (Kelchtermans, 1996; Santoro, 2011; Farouk, 2012).

## Conclusion, Implications, Limitations, and Further Research Directions

This article makes three contributions to the literature. First, it extends the application of the emergent sociological perspective of inhabited institutionalism to research on teachers’ emotions. Second, it uses that perspective to advance our understanding of the dynamic nature of the construction of teachers’ emotions. It suggests that teachers’ emotions are an interactional–institutional construction rather than being constructed purely by institutions or meaning-making. Third, it illustrates that the construction of teachers’ emotions involves a dynamic relationship between institutional logics, the institutional context of school organizations, and social actors, such as school administrators and teachers, engaging in the negotiation of meaning. It thus offers an alternative framework that enables us to understand how teachers’ emotions are dynamically constructed by institutions and interactions, especially in the context of education reforms.

If teachers’ emotions are an interactional–institutional construction, two approaches can be recommended to education policy makers and school administrators to improve teachers’ emotions. The first is an institutional approach. As the findings show, the institutional logic of accountability has become dominant in the institutional environment of education and has replaced the instructional meaning of education with managerial meanings. This makes it difficult for teachers to identify with the meaning of their work and to feel a connection with their work, thus generating negative emotions. Therefore, policy makers and school administrators should de-emphasize educational accountability, effectiveness, efficiency, and economy in a managerial sense in policy initiatives and school management. This may institutionally empower teachers to perform instructional work and to discover meaning in their work. The second is an interactional approach. Policy makers and school administrators should create opportunities to interact with teachers to allow for negotiation over the meanings of education, teaching, and learning. Such negotiation may facilitate teachers in developing a better understanding of their current situations, resulting in them assigning more positive meaning to their work and in turn generating positive emotions. As teachers’ emotions are an interactional–institutional construction, making changes in institutions may induce changes in interactions and vice versa. In other words, policy makers and school administrators can apply either an institutional or interactional approach in attempting to improve teachers’ emotions.

One limitation of the present study is that it only focuses on the institutional context of secondary schools in Hong Kong. To some extent, the institutional context of secondary schools may differ from that of primary schools. Thus, the interactional–institutional construction of teachers’ emotions in primary schools may have different features to those identified by the study. Future studies are recommended to apply the perspective of inhabited institutionalism to investigate primary school teachers’ emotions in or outside Hong Kong to enrich our understanding of the construction of teachers’ emotions. Additionally, although the study indicates an asymmetric relationship between the institutional logics of whole-person education and accountability, there may be other possible relationships between institutional logics that have not been identified. However, due to limited resources, the present study has not examined these relationships. Thus, future studies should innovatively apply various methods of collecting and analyzing different kinds of historical, observational, interview, and artificial data to investigate what institutional logics coincide in institutional environments of education, how they interact with one another, how these interactions influence the institutional context of schools and are inhabited by social actors who negotiate meaning, and how the interactions affect teachers’ emotions.

## Data Availability Statement

The datasets generated for this study are available on request to the corresponding author.

## Ethics Statement

The studies involving human participants were reviewed and approved by the Human Research Ethics Committee (HREC), The University of Hong Kong. The patients/participants provided their written informed consent to participate in this study.

## Author Contributions

The author confirms being the sole contributor of this work and has approved it for publication.

## Conflict of Interest

The authors declare that the research was conducted in the absence of any commercial or financial relationships that could be construed as a potential conflict of interest.
